# Effectiveness and cost-effectiveness of web-based treatment for phobic outpatients on a waiting list for psychotherapy: protocol of a randomised controlled trial

**DOI:** 10.1186/1471-244X-12-131

**Published:** 2012-08-31

**Authors:** Robin N Kok, Annemieke van Straten, Aartjan Beekman, Judith Bosmans, Manja de Neef, Pim Cuijpers

**Affiliations:** 1 Department of Clinical Psychology and the EMGO institute for Health and Care Research, Faculty of Psychology and Education, VU University Amsterdam, Van der Boechorststraat 1, 1081, BT, Amsterdam, The Netherlands; 2 Department of Psychiatry and the EMGO institute for Health and Care Research, VU University Medical Centre, P.O. Box 7057, 1007, MB, Amsterdam, The Netherlands; 3 Department of Health Sciences and the EMGO Institute for Health and Care Research, Faculty of Earth and Life Sciences, VU University Amsterdam, De Boelelaan 1085, 1081, HV, Amsterdam, The Netherlands; 4 Dutch Association of Cognitive Behaviour Therapy, Utrecht, The Netherlands

**Keywords:** Phobias, Phobic disorders, Web-based intervention, Internet therapy, Randomised controlled trial, Cost-effectiveness, Outpatients

## Abstract

**Background:**

Phobic disorders are highly prevalent and constitute a considerable burden for patients and society. As patients wait for face-to-face psychotherapy for phobic disorders in outpatient clinics, this time can be used for guided self-help interventions. The aim of this study is to investigate a five week internet-based guided self-help programme of exposure therapy in terms of clinical effectiveness and impact on speed of recovery in psychiatric outpatients, as well as the cost-effectiveness of this pre-treatment waiting list intervention.

**Methods/design:**

A randomised controlled trial will be conducted among 244 Dutch adult patients recruited from waiting lists of outpatient clinics for face-to-face psychotherapy for phobic disorders. Patients suffering from at least one DSM-IV classified phobic disorder (social phobia, agoraphobia or specific phobia) are randomly allocated (at a 1:1 ratio) to either a five-week internet-based guided self-help program followed by face-to-face psychotherapy, or a control group followed by face-to-face psychotherapy. Waiting list status and duration are unchanged and actual need for further treatment is evaluated prior to face-to-face psychotherapy. Clinical and economic self-assessment measurements take place at baseline, post-test (five weeks after baseline) and at 3, 6, 9 and 12 months after baseline.

**Discussion:**

Offering pre-treatment internet-based guided self-help efficiently uses time otherwise lost on a waiting list and may increase patient satisfaction. Patients are expected to need fewer face-to-face sessions, reducing total treatment cost and increasing speed of recovery. Internet-delivered treatment for phobias may be a valuable addition to psychotherapy as demand for outpatient treatment increases while budgets decrease.

**Trial registration:**

Netherlands Trial Register NTR2233

## Background

Anxiety disorders have a pooled lifetime prevalence of 19.8% [1]. This makes these disorders the most prevalent mental disorders for women, and the second most prevalent for men. Specific phobias are the most common form of anxiety disorders for both genders, with a total 12-month prevalence of 7.1%, followed by social phobia (4.8%) and agoraphobia without panic disorder (1.2%). Phobias are characterised by an excessive fear of situations or objects and are often treated by gradual exposure to the fear-inducing stimulus and/or cognitive behavioural therapy.

The economic burden of these conditions is considerable [2], as is the negative impact on quality of life and psychosocial functioning [3], especially if the patient is often confronted with the fear-inducing situation or object in life. In clinical populations this burden may have long-term effects on psychosocial functioning and well-being, as research suggests that often more than a decade passes between onset of symptoms and first therapy attendance [4]. The prevalence and burden of phobic disorders call for effective and cost-effective treatment options and the Internet offers an accessible and widespread platform to disseminate low-threshold treatment for phobias.

Research has shown that guided self-help is effective for anxiety disorders [5] and that it can be as effective as face-to-face (FTF) psychotherapy [6]. Self-help programs adapted for the Internet have shown similar results [7,8], e.g. for social anxiety disorder [9], generalised anxiety disorder [10] and severe health anxiety [11]. The use of internet interventions for treating anxiety disorders has been well-established and deemed useful and acceptable in primary care and the general population [12]. A recent small scale study found a large effect size of internet-based treatment for panic disorder in a psychiatric outpatient setting [13] and internet-based cognitive behavioural therapy (CBT) for adolescent anxiety appeared to be as efficacious as clinic-based FTF psychotherapy [14]. Apart from these studies, little is known about the clinical effectiveness or cost-effectiveness of internet-based CBT in psychiatric outpatient settings. Few studies have examined internet-based treatment of phobias in particular and these tended to focus on social phobia in the general population or primary care patients.

Waiting lists of varying lengths are ubiquitous in psychiatric outpatient clinics. As demand for psychotherapy often exceeds supply, patients cannot be treated immediately. Time otherwise ‘lost’ while a patient is on a waiting list could be spent more efficiently when a waiting list guided self-help intervention is offered that comprises elements common in FTF psychotherapy for phobias, e.g. psychoeducation, goal setting and exposure exercises. Offering patients a self-help intervention while on a waiting list for FTF treatment can be advantageous to both outpatient clinics and patients for several reasons.

Firstly, following the stepped-care model, the intervention may be sufficient treatment for some phobic disorders, cancelling the need for further psychotherapy and preventing inappropriate use of outpatient resources.

Secondly, being placed on a waiting list may be disappointing to patients who have been suffering from a phobia for a long time. This disappointment may lead to pre-treatment attrition, i.e., patients not starting FTF psychotherapy or 'no-show'. This has been shown to be relatively high in FTF settings, up to 30% in social phobia [15]. Offering the intervention during the waiting period offers a mutual benefit of keeping the patient actively engaged in his or her treatment and thus possibly reducing this pre-treatment attrition.

Thirdly, depending on the uptake and effects of the guided self-help intervention, fewer FTF psychotherapy sessions might suffice when the waiting period has ended and FTF treatment is scheduled, thus lowering the pressure on psychiatric outpatient clinics. This can also lead to lower per-patient treatment costs.

As healthcare budgets stagnate or decline and demand for treatment for phobias increases, a larger number of patients will need to be treated at lower costs per patient.

### Aims and hypotheses

The objective of this study is to establish the clinical and cost-effectiveness of an internet-based brief guided self-help intervention for phobic patients in outpatient clinics. We expect that patients are more satisfied as they can start treatment while on a waiting list and that outpatient clinics can reduce the number of costly FTF psychotherapy sessions due to the skills and knowledge already acquired by the patient in the guided self-help intervention. Furthermore, we expect that patients in the intervention group will recover more quickly than in the control group.

## Methods/design

### Study design

This study is a randomised controlled trial alongside an economic evaluation. Patients on a waiting list for FTF psychotherapy are randomised to either the intervention group, who will receive access to the internet-based guided self-help program, or the control group who will receive a self-help book for motivational purposes without additional guidance. Both groups will receive FTF psychotherapy at the outpatient clinic following the guided self-help or unguided motivational book.

The study protocol, information brochure, questionnaires and informed consent form were approved by the Medical Ethics Committee of the VU University Medical Centre (registration number 2010/77).

### Inclusion and exclusion criteria

All patients aged 18 and over on the waiting list of participating outpatient clinics and willing to enrol in an internet-based self-help program are eligible for this study. Inclusion criteria are: suffering from a DSM-IV diagnosis of agoraphobia, social phobia or specific phobia as determined by a trained interviewer using a diagnostic interview (the Composite International Diagnostic Interview (CIDI) [16]). Patients are allowed to continue their psychotropic medication if they stay on a stable regimen throughout the intervention. Exclusion criteria are: no access to the internet, inadequate self-reported computer literacy, inadequate proficiency in Dutch, suicidal ideation or increased risk of suicide and bipolar or psychotic disorder. Comorbid disorders other than bipolar or psychotic disorders are allowed.

### Recruitment

Patients are recruited at several outpatient clinics. For practical reasons, only clinics with a high number of monthly enrolments were selected. Generally speaking, patients are referred to the outpatient clinic by their general practitioner (GP). At the clinic the patient is briefly screened and placed on a waiting list. After some time an initial meeting with a therapist takes place, during which the patient’s treatment needs and preferences are determined in detail. Based on this, a treatment suited to the individual patient is selected, e.g. group CBT therapy for social anxiety disorders or individual exposure-based therapy for specific phobias. The researchers are not involved in this decision process. Depending on therapist workload, treatment modality and other factors, there is considerable variability in the length of the interval between referral to the clinic and first therapeutic contact. This interval typically ranges from five - twenty weeks.

**Figure 1:**
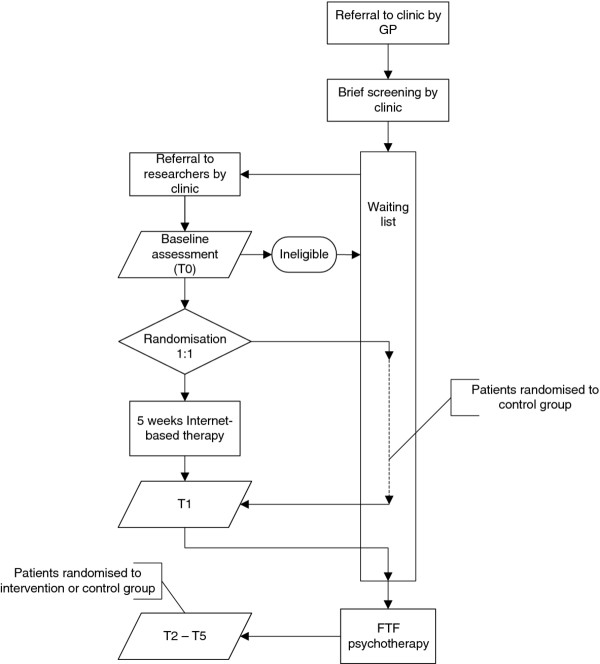
Trial flowchart.

For this study, patients are recruited when placed on the post-screening waiting list of outpatient clinics. Patients agreeing to be contacted by the researchers are approached by the researchers and a telephone diagnostic interview (CIDI) is conducted by a research assistant to determine clinical status. If eligible, the patients are administered a telephone baseline assessment and randomised. Patients are sent an information kit containing a study brochure, informed consent form and stamped return envelope. The first therapeutic contact (i.e., first meeting with therapist to determine patient’s status and needs) is not postponed if the patient is enrolled in this study. For a detailed representation of participant flow in this study, see Figure 1.

### Randomisation

After baseline assessment patients are randomised by an external researcher using a computer-generated randomisation chart which is stratified by clinic. Block randomisation is used with eight allocations per block, using a 1:1 ratio. Researchers are blind to the randomisation procedure and to treatment group allocation until randomisation is complete and unequivocal. Due to the nature of the intervention, patients cannot be blinded to group allocation as the two treatment options need to be revealed prior to randomisation.

### Intervention: internet-based guided self-help exposure therapy

The intervention is adapted for the internet from a self-help book on phobias [17], which is based on exposure therapy, the de facto standard treatment in phobic anxiety disorders. The internet treatment is offered at no cost to the patient, takes five weeks to complete and is based on psycho-education and exposure therapy with optional though schedules. Patients select fear-inducing situations or stimuli and construct a ‘fear hierarchy’ to increasingly expose themselves to these situations or stimuli in gradual, ascending steps. Background information and psychoeducation on phobias is given, as well as information on relapse prevention, recommendations and gathering support from friends. A number of example patients are included to illustrate the principles and concepts of the intervention. Support consists of online coaching messages, delivered through a closed, secured message system on the intervention website. This is offered by master’s level students of clinical psychology who have received training in online coaching. Research has shown that relatively inexperienced persons can provide effective online coaching on a level equal to an experience clinician, if they are adequately trained, supervised and the treatment is sufficiently standardized and manualized [18,19]. The patient completes exposure exercises as homework assignments and reports on his or her accomplishments each week. The coach monitors the fear hierarchy and evaluates whether the patient’s exposure exercise planning is feasible, relevant and useful and replies with a support message with praise, recommendations and information relevant to the patient’s homework accomplishments or experiences.

The intervention is tunnelled, i.e., no new material is available to the patient until the patient has reported on that week’s achievements and the coach has provided feedback on these achievements. If applicable, the coach sends a standardized reminder message if the patient did not use the website that week. Material from previous weeks remains accessible to the patient. To allow for any technical difficulties, patients are allowed to overrun the five-week intervention by one week.

All coaching will be supervised by an experienced psychotherapist (MdN), who is trained in in-vivo exposure therapy. We will keep close contact with the institutions and therapists and carefully monitor each patient, sending the therapist an informative reminder message of the patient’s participation in the study immediately before first therapeutic contact. This is to encourage the therapist to assess the patient’s condition first and, if deemed necessary, to adjust therapeutic approach and aims to suit the patient’s prior knowledge and previous exposure accomplishments attained during the intervention.

### Control group

The control group will receive a self-help book [17] free of charge, without additional support. This self-help book contains the same information as the intervention website, i.e., psycho-education and exposure therapy, but has more optional exercises such as thought schedules. It was deemed unethical to enrol patients into the trial and subsequently allocate them to a control condition without offering at least some compensation for their time and effort. The book is sent as-is, without further instructions or expectations, as unguided bibliotherapy. The patients will have no contact with the research team with regards to the self-help book during or after the self-help period.

### Psychotherapy

Patients in both the intervention group and the control group are scheduled to receive follow-up FTF psychotherapy by licensed health care psychologists at the end of the waiting list but patients can choose to decline further FTF treatment.

### Assessments

In total, 6 assessments will take place. The baseline assessment takes place before randomisation, with follow-up assessments post-intervention (at five weeks), and at 3, 6, 9 and 12 months. Baseline measures are collected by telephone to ensure pre-treatment assessments are present for all patients and follow-up measures are administered as online questionnaires.

### Primary outcome: presence of phobia and avoidance behaviour

The primary outcome measure is the Fear Questionnaire (FQ) [20]. This instrument measures severity of fear and avoidance of phobic stimuli. The FQ is a self-rated questionnaire adapted for use on the internet and consists of 15 items that measure total phobia and five items that measure anxiety and depression. Patients are asked to rate how often they avoid different situations on a scale of 0 (“I do not avoid this”) to 8 (“I always avoid this”).

Due to the large number of possible specific phobias, the primary phobia for which the patient seeks treatment is filled out in a single item rather than chosen from a list.

A total phobia score ranges from 15–120 and is calculated from three subscales; agoraphobia, blood/injection phobia and social phobia. A higher score indicates greater avoidance of phobic stimuli. A single item measures global phobic distress, or the extent to which phobic symptoms limit the patient’s daily activities, on a scale of 0 (no phobia) to 8 (extremely limiting or disruptive). The psychometric validity of the FQ has been established for both the original [21,22] and the Dutch version [23].

### Secondary outcomes

#### Anxiety

The Beck Anxiety Inventory (BAI) [24] is a 21-item self-report questionnaire which focuses on physiological manifestations of anxiety, e.g. sweating, trembling and increased heart rate. Items can be scored from 0–3 for a total sum score range of 0–63, where higher scores indicate higher levels of self-reported manifestations of anxiety. The BAI has been validated for patients with agoraphobia [25] and other anxiety disorders [26].

### Depressive symptoms

The Centre for Epidemiological Studies Depression Scale (CES-D) [27] is administered as a self-rated questionnaire on the internet. The CES-D consists of 20 items which are scored from 0–3, the total score can range from 0–60, where a higher score indicates a higher level of depressive symptoms. A Dutch version of the CES-D has been validated in an internet-administrated form [28].

### Quality of life

Health-related quality of life is measured using an internet-based version of the EuroQol (EQ-5D). The EQ-5D appears to have sufficient validity [29,30]. Patients report whether they have ‘no problems’, ‘some problems’ or ‘extreme problems’ on each of five domains: mobility, self-care, daily activities, pain and discomfort and anxiety/depression. The combination of items results in 243 distinct health states. This results in a utility score for each health state that ranges from death (0) to perfect health (1). The Dutch tariff will be used to value these health states [31].

### Treatment satisfaction

Satisfaction with the internet intervention or the self-help book is evaluated using the Client Satisfaction Questionnaire (CSQ-8) [32] which has been validated for use in a Dutch population [33]. The CSQ-8 has been adapted to a self-rated internet-administered questionnaire. A total of 8 items can be scored from 1–4, yielding a total range score of 8–32, where a higher score indicates a higher level of satisfaction with received care.

Several items are added to assess the patient’s satisfaction with the intervention website and coach or the self-help book and, if applicable, why the patient decided to stop using the book or the website.

### Alcohol use

The CAGE questionnaire [34] is a brief screening measure for alcohol dependence. It has been validated as being able to establish heavy drinking and/or alcohol dependence [35]. Although heavy alcohol use is not among the exclusion criteria, we monitor problematic alcohol use in the light of the complex relationship between anxiety disorders and alcohol consumption [36].

### Health care utilization and work absenteeism

The iMTA Questionnaire on Costs Associated with Psychiatric Illness (TiC-P) [37] is a structured questionnaire to measure costs from a societal perspective. Cost categories that are included are use of medication, health services and informal care (direct costs), and work absenteeism or and/or reduced work productivity (indirect costs). The friction cost method will be applied to estimate costs of work absenteeism. Dutch standard costs will be used to value direct and indirect costs [38].

### Data on FTF psychotherapy after intervention or self-help book

Data on received psychotherapy at the outpatient clinic will be retrieved post-hoc from the institutions’ electronic medical records for both groups. Data on the modality of psychotherapy (e.g. individual CBT, group therapy) and the number of sessions will be recorded.

### Internet-specific data and sundry expenses

Internet usage specifics such as number of logins, total duration of session, number of modules completed will be collected on a per-patient basis. Total costs for development, maintenance, patient screening, coaching and sundry expenses are calculated on a per-patient basis (Table 1).

**Table 1:** Measurements collected

	**Baseline/T0**	**Post-treatment (5 weeks)**	**3 months**	**6 months**	**9 months**	**12 months**
CIDI	x					
FQ	x	x	x	x		x
BAI	x	x	x	x		x
CES-D	x	x	x	x		x
CSQ		x	x			
EQ5D	x	x	x	x	x	x
TiC-P	x	x	x	x	x	x
Satisfaction		x				
CAGE	x	x	x	x		x

### Sample size

To obtain 90% statistical power with a 2-sided α equal to 0.05 and assuming a mean standardised effect size (Cohen’s *d*) of 0.7 in the intervention group and 0.2 in the control group, 85 patients are needed for each trial arm to establish the clinical effect of the internet intervention compared to the waiting list condition (170 patients in total). We assume a dropout rate of 30% during one-year follow-up, thus, 244 patients should be included.

### Statistical analyses

Results will be reported on both intention-to-treat and per-protocol basis. Missing values will be imputed using multiple imputation, as recent research suggests that this is a reliable method for dealing with missing values within datasets [39].

### Economic outcomes

The mean difference in costs between the control group and intervention group is calculated, using bootstrapping to estimate 95% confidence intervals. Incremental cost-effectiveness ratios (ICERs) will then be calculated by dividing the difference in costs between the two groups by the difference in clinical effects. Bootstrapping will be used to estimate the uncertainty surrounding the ICERs, which will be graphically represented on cost-effectiveness planes. Cost-effectiveness acceptability curves and net monetary benefits will also be calculated. Cost-effectiveness acceptability curves show the probability that the intervention is cost-effective in comparison with usual care for a range of different ceiling ratios, thereby showing decision uncertainty [40].

## Discussion

This study will evaluate the clinical and cost-effectiveness of offering internet-based guided self-help for phobic outpatients. Results of internet therapy for phobias in the general population are encouraging and implementation in outpatient clinics may be a valuable addition to existing treatment options. Also, offering of internet therapy while a patient is waiting for FTF psychotherapy makes efficient use of this time and may have large benefits for both the patient and the outpatient clinic in terms of clinical effectiveness, cost-effectiveness and patient satisfaction.

This study faces a number of challenges. Firstly, it is imperative that the patients’ therapists assess the needs of their patients prior to actually starting FTF psychotherapy. If the therapist starts the patient on a standard treatment (e.g. a fixed number of FTF psychotherapy sessions as prescribed by treatment protocol) regardless of the skills and knowledge acquired during the internet intervention, then the addition of the internet intervention would be ineffectual in lowering treatment costs.

Secondly, there is considerable variability in the time patients spend on a waiting list. This varies both between and within institutions and is influenced by several factors such as seasonal changes and peaks or drops in patient referrals or staff workloads. The direct implication is that there is some variability in the time between the end of the guided self-help intervention and the start of FTF psychotherapy. Patients are allowed to re-visit the intervention website throughout the remainder of the waiting list period. However, they will no longer be able to communicate with a coach. Since patients in both the intervention group and the control group are on the same waiting list, there is no reason to assume that there is a difference in time spent on the waiting list between both groups.

Thirdly, attrition from and lack of adherence to internet-based interventions are debated topics in internet-based interventions. A recent meta-analysis found a mean dropout rate of 31% for internet-based interventions [41]. These dropout figures are often seen as major drawbacks for the implementation of internet-based interventions. However, it should be noted that a meta-analysis of 123 studies found a mean non-usage attrition in psychotherapy of 47% [42]. Two main categories of attrition are patients not or only partially using the intervention (non-usage attrition) and patients not completing follow-up assessments (dropout attrition [43]). Recently, a systematic review of FTF treatments for social phobia found no link between pre-treatment variables and non-usage attrition [44]. All aforementioned studies on attrition have focussed mainly on the general population instead of a clinical (outpatient) population. To prevent non-usage and dropout attrition, several e-mail and telephone reminders are scheduled throughout this trial. Also, we assume dropout in this outpatient population to be lower than that of internet interventions that recruit from the general population.

To our knowledge, this is the first large-scale pragmatic cost-effectiveness and effectiveness study of an internet-based intervention for anxiety in outpatient clinics. Considering the increase in the addition of e-mental health interventions to the treatment packages offered at psychiatric outpatient clinics, the findings of this study will provide valuable information on the additional value of online treatment of anxiety disorders in general and phobias in particular.

## Competing interests

The author(s) declare that they have no competing interests.

## Authors’ contributions

PC and MdN authored the intervention. PC and AvS drafted the study protocol. RK authored this article. MdN provides professional supervision for students supporting the patients. AvS, JB, PC, MdN and AB provided feedback and suggestions for this manuscript. All authors read and approved the final manuscript.

## Pre-publication history

The pre-publication history for this paper can be accessed here:

http://www.biomedcentral.com/1471-244X/12/131/prepub
